# Diagnostic value of saline contrast sonohysterography comparing with hysteroscopy for detecting endometrial abnormalities in women with abnormal uterine bleeding

**Published:** 2011

**Authors:** Mohammad Ali Karimzadeh, Razieh Dehghani Firouzabadi, Farzaneh Goharzad

**Affiliations:** 1 Department of Obstetrics and Gynecology, Research and Clinical Center for Infertility, Shahid Sadoughi University of Medical Sciences, Yazd, Iran.; 2 Department of Obstetrics and Gynecology, Shahid Sadoughi Hospital, Shahid Sadoughi University of Medical Sciences, Yazd, Iran.

**Keywords:** *Abnormal uterine bleeding*, *Uterine abnormalities*, *Sonohysterography*, *Hysteroscopy*

## Abstract

**Background:**

Abnormal uterine bleeding is a common presentation of uterine abnormalities among premenopausal and postmenopausal women.

**Objective:**

To evaluate and compare the diagnostic accuracy of saline contrast sonohysterography and hysteroscopy for detecting the cause of abnormal uterine bleeding.

**Materials and Methods:**

A total of 65 women with abnormal uterine bleeding were enrolled in this study. A prior saline contrast sonohysetrography followed by a hysteroscopy was performed in all cases. Sensitivity, specificity, positive and negative predictive value and test accuracy were calculated.

**Results:**

As the most common abnormality, SCSH showed hyperplasia in 19 patients while hysteroscopy diagnosed polyp in 15 cases. A sensitivity of 73.3%, 71.4% and 90.9% were reported for polyp, hyperplasia and submucous myoma respectively whereas the specificity was calculated 96% for polyps, 82.3% for hyperplasia and 90.7% for submucous myoma.

**Conclusion:**

Comparing with hysteroscopy, sonohysterography showed a high sensitivity and specificity for detecting submucous myoma but not for endometrial polyp and endometrial hyperplasia.

## Introduction

Abnormal uterine bleeding () is a common presenting symptom among women. AUB is presented in 33% of women referred to gynecologists and this pattern increases to 69% in premenopausal and post menopausal women (1). AUB can be caused by a variety of uterine abnormalities such as polyp, submucous myoma, endometrial hyperplasia and endometrial carcinoma. Cases of AUB require a good diagnostic and therapeutic approach which can be acquired by traditional dilatation and curettage or recent and more effective diagnostic tools.

A variety of tools can be used for the diagnosis of uterine abnormalities lead to AUB. Among them, transvaginal sonography (TVS), saline contrast sonohysterography (SCSH) and hysteroscopy have been used commonly. TVS is the first line investigation tool for diagnosis of uterine abnormalities, whereas hysteroscopy has become the gold standard for the evaluation of patients with AUB. In postmenopausal women TVS is an effective screening test for evaluation of abnormal uterine bleeding caused by endometrial atrophy (2). But in the figure of thickened and inhomogeneous endometrium, TVS is presented as a low specificity and limited diagnostic test which can be replaced by SCSH (3, 4). SCSH can distinguish between focal lesions such as polyps and submucous myomas (5, 6) and diffuse lesions like hyperplasia and cancer accurately (7, 8). Furthermore, hysteroscopy is an effective but expensive and invasive screening test for evaluation of the uterine cavity in both pre and postmenopausal women with AUB (9, 10).

Preoperative imaging of the uterine cavity is very important and the results can be necessary for the surgical management. A useful imaging technique for accurate diagnosis should be highly sensitive and specific, non invasive and cost-effective. It seems that SCSH is a non invasive, cheap and feasible technique with lower pain. In order to compare SCSH and hysteroscopy, the majority of women found that SCSH was not painful, whereas only 25% said the same for hysteroscopy (11). If it can be proven that the sensitivity and specificity of SCSH and hysteroscopy are the same, it can be recommended as the first line detecting tool for uterine abnormalities caused AUB.

The objective of this study was to evaluate the sensitivity and specificity of SCSH compare with hysteroscopy in the investigation of women of reproductive age presenting with AUB.

## Materials and methods

**Patients**

65 consecutive women presenting AUB or infertility were enrolled in this diagnostic study. These patients were referred to both Yazd Shahid Sadoughi and Yazd Madar Hospital from March 2006 to February 2007. The women who have any evidence of systemic disease such as diabetes, hypertension, PCO, thyroid disease, evidence of pregnancy, evidence of pelvic inflammatory disease and history of uterine surgeries were excluded from the study. After obtaining informed consent, saline contrast sonohysetrography followed by a hysteroscopy was done in all cases. The institutional Review Board at Yazd University of Medical Sciences approved this prospective study.

**Imaging techniques**

Regarding SCSH, a sterile speculum was passed, the cervix visualized and disinfected with Betadine solution. A flexible Foley catheter number 8 with inflatable balloon (Supa, Tehran, Iran) was inserted through the cervical canal into the uterine cavity. After confirmation of the position of the catheter, 10ml of 0.9% sterile saline solution was injected into the uterine cavity slowly and continued to obtain optimal views of endometrial cavity. By using concomitant transvaginal sonography, the uterine cavity was evaluated for detecting any abnormality or pathological condition. This procedure was performed by a single investigator without the use of local anesthesia or prophylactic antibiotic therapy. All patients had diagnostic operative hysteroscopy under a general anesthesia. Hysteroscopy was performed using cervix dilatation, 2 Misoprostol tablet (6 hours before operation) and prophylactic antibiotic. The hysteroscopies were done by the expert operator who was blinded to the SCSH results. Endometrial biopsy was carried out directly after hysteroscopy.

**Statistical analysis**

The Statistical Package for the Social Sciences 13.0 software was used to analyze data of all patients. Sensitivity, specificity, positive and negative predictive value and test accuracy were calculated for SCSH as compared with findings of hysteroscopy. 

## Results

Among 65 evaluated women, 78.5% presented AUB and 21.5% had infertility problem. The mean age of women presenting AUB was 37.02±7.85 years and infertile women had mean age of 25.50± 4.22 years. The most common abnormality in SCSH was hyperplasia (29.2%) while it was polyp (23.1%) in hysteroscopy. Hyperplasia was detected in 21.5% of cases by hysteroscopy and polyp was seen in 20% of patients using SCSH. As the second cause, SCSH suggested the presence of cancer in 23.1% of women whereas it was hyperplasia among 21.5% of cases in hysteroscopy group. The number and percentage of abnormalities detected in patients are listed in table I. According to hysteroscopy results the diagnosis of 36.9% of women remained unknown and it was 26.2% in SCSH. SCSH showed a sensitivity of 73.3%, 71.4% and 90.9% for polyp, hyperplasia and submucous myoma respectively whereas the specificity was reported 96% for polyps, 82.3% for hyperplasia and 90.7% for submucous myoma. Table II shows the sensitivity, specificity, positive predictive value (PPV), negative predictive value (NPV) and accuracy for SCSH compared with hysteroscopy as a gold standard.

**Table I:** Number of uterine abnormalities diagnosed using SCSH and hysteroscopy

	**SCSH ** **no (%)**	**Hysteroscopy no (%)**
Polyps	12(20%)	15(23.1%)
Hyperplasia	19(29.2%)	14(21.5%)
Submucous myoma	1(1.5%)	1(1.5%)
Cancer	15(23.1%)	11(16.9%)
Unknown	17(26.2%)	24(36.9%)

**Table II:** Sensitivity, specificity, positive predictive value, negative predictive value and accuracy for SCSH compared with hysteroscopy.

	**Sensitivity**	** Specificity**	**Positive predictive value**	**Negative predictive value **	**Accuracy **
Polyps	73.3%	96%	84.6%	92.3%	90.7%
Hyperplasia	71.4%	82.3%	52.6%	91.3%	90%
Submucous myoma	90.9%	90.7%	66.7%	98%	90.7%

## Discussion

There are different methods for detecting causes of AUB as a common chief complain in premenopausal or post menopausal women. For many years, dilatation and curettage was performed as a first line approach, because the sonographical tools have limited accuracy specially unavailability of endometrial sampling (12, 13). Nowadays, this limitation has been overcome by TVS and SCSH followed by hysteroscopy and endometrial sampling. SCSH is a new evaluating method that makes distension in uterine cavity to visualize endometrial surface (14). In addition it has less pain in patients with minimal cost, and performs easier and faster with more safety comparing with hysteroscopy (15, 16). In this research we compared SCSH as an accurate method to distinguish local and diffuse lesions (5, 6) with hysteroscopy as a gold standard diagnostic method.According to our study, in detecting submucous myoma, SCSH has a good sensitivity of 90.9 and specificity of 90.7 compared with hysteroscopy. Regarding polyp and endometrial hyperplasia, hysteroscopy presented more sensitivity and specificity than SCSH. Some studies reported a similar diagnostic accuracy (17-20). One study found a sensitivity of 100% and specificity of 98.3% for myoma versus sensitivity of 87.5% and specificity of 95.9% for polyp; using sonohysterography compared with hysteroscopy as the reference test (17). In Kelekci *et al* study, for detecting endometrial polyp using saline infusion sonography; sensitivity, specificity, PPV and NPV were 70%, 100%, 100% and 90.9% retrospectively whereas all of these parameters were 100% in detecting submucous myoma (18). The current study showed that sonohysterography is less sensitive and specific for detecting polyp and endometrial hyperplasia than hysteroscopy. In disagreement with our results, Soares *et al* indicated that sonohysterography had 100% sensitivity, 100% PPV and 100% diagnostic accuracy for endometrial polyps, fibroids and endometrial hyperplasia (19). In addition, Nanda *et al* reported that there is no missing in diagnosis of endometrial polyp using sonohysterography (21). In one study, 135 patients with AUB and subfertility were evaluated and the result showed that SCHS is a very accurate method for detecting focal endometrial pathology, compared to diagnostic hysteroscopy (22). Another study claimed that hysteroscopy can be replaced by Saline-infusion sonography in more than half of AUB cases (23) and also there is very good agreement between sonohysterography and hysteroscopy for diagnosis endometrial abnormalities in postmenopausal women (24). Mathew *et al* (25) concluded that saline infusion sonohysterography is a simple evaluating method with minimal invasiveness and cost which is more accurate than TVS and can be done as a screening tool before hysteroscopy. Saline infusion sono-hysterography also is a satisfactory method of identifying endometrial lesions which is less invasive alternative to hysteroscopy and result in less morbidity in the evaluation of AUB in women (26). 

## Conclusion

According to our result, comparing with hysteroscopy; sonohysterography is sensitive and specific for diagnosis of submucous myoma but not for endometrial polyp and endometrial hyperplasia. However hysteroscopy is a therapeutic procedure and it is preferable for its therapeutic role. 

## Note

***This article extracted from residential thesis***
